# Predicting metabolic syndrome by obesity- and lipid-related indices in mid-aged and elderly Chinese: a population-based cross-sectional study

**DOI:** 10.3389/fendo.2023.1201132

**Published:** 2023-07-28

**Authors:** Yuqing Li, Jiaofeng Gui, Haiyang Liu, Lei-lei Guo, Jinlong Li, Yunxiao Lei, Xiaoping Li, Lu Sun, Liu Yang, Ting Yuan, Congzhi Wang, Dongmei Zhang, Huanhuan Wei, Jing Li, Mingming Liu, Ying Hua, Lin Zhang

**Affiliations:** ^1^ Department of Graduate School, Wannan Medical College, Wuhu, An Hui, China; ^2^ Health Center, Wannan Medical College, Wuhu, An Hui, China; ^3^ Department of Surgical Nursing, School of Nursing, Jinzhou Medical University, Jinzhou, Liaoning, China; ^4^ Department of Occupational and Environmental Health, Key Laboratory of Occupational Health and Safety for Coal Industry in Hebei Province, School of Public Health, North China University of Science and Technology, Tangshan, Hebei, China; ^5^ Obstetrics and Gynecology Nursing, School of Nursing, Wannan Medical College, Wuhu, An Hui, China; ^6^ Department of Emergency and Critical Care Nursing, School of Nursing, Wannan Medical College, Wuhu, An Hui, China; ^7^ Department of Internal Medicine Nursing, School of Nursing, Wannan Medical College, Wuhu, An Hui, China; ^8^ Department of Pediatric Nursing, School of Nursing, Wannan Medical College, Wuhu, An Hui, China; ^9^ Department of Surgical Nursing, School of Nursing, Wannan Medical College, Wuhu, An Hui, China; ^10^ Rehabilitation Nursing, School of Nursing, Wannan Medical College, Wuhu, An Hui, China

**Keywords:** metabolic syndrome, cross-sectional study, middle-aged and elderly, receiver operating characteristic curve, ROC (receiver operating characteristic curve)

## Abstract

**Objective:**

To predict the optimal cut-off values for screening and predicting metabolic syndrome(MetS) in a middle-aged and elderly Chinese population using 13 obesity and lipid-related indicators, and to identify the most suitable predictors.

**Methods:**

The data for this cross-sectional investigation came from the China Health and Retirement Longitudinal Study (CHARLS), including 9457 middle-aged and elderly people aged 45-98 years old. We examined 13 indicators, including waist circumference (WC), body mass index (BMI), waist-height ratio (WHtR), visceral adiposity index (VAI), a body shape index (ABSI), body roundness index (BRI), lipid accumulation product index (LAP), conicity index (CI), Chinese visceral adiposity index (CVAI), triglyceride-glucose index (TyG-index) and their combined indices (TyG-BMI, TyG-WC, TyG-WHtR). The receiver operating characteristic curve (ROC) was used to determine the usefulness of indicators for screening for MetS in the elderly and to determine their cut-off values, sensitivity, specificity, and area under the curve (AUC). Association analysis of 13 obesity-related indicators with MetS was performed using binary logistic regression analysis.

**Results:**

A total of 9457 middle-aged and elderly Chinese were included in this study, and the overall prevalence of the study population was 41.87% according to the diagnostic criteria of NCEP ATP III. According to age and gender, the percentage of males diagnosed with MetS was 30.67% (45-54 years old: 30.95%, 55-64 years old: 41.02%, 65-74 years old: 21.19%, ≥ 75 years old: 6.84%). The percentage of females diagnosed with MetS was 51.38% (45-54 years old: 31.95%, 55-64 years old: 39.52%, 65-74 years old: 20.43%, ≥ 75 years old: 8.10%). The predictive power of Tyg-related parameters was more prominent in both sexes. In addition, LAP and CVAI are also good at predicting MetS. ABSI had a poor prediction ability.

**Conclusions:**

Among the middle-aged and elderly population in China, after adjusting for confounding factors, all the indicators except ABSI had good predictive power. The predictive power of Tyg-related parameters was more prominent in both sexes. In addition, LAP and CVAI are also good at predicting MetS.

## Introduction

Metabolic syndrome(MetS) (1, 2) refers to an energy disorder characterized by the presence of at least any three or more of the following risk factors: elevated fasting glucose, elevated blood pressure, elevated triglyceride levels, reduced high-density lipoprotein cholesterol (HDL-C), and obesity (especially central obesity). A study by scholars Ranasinghe et al. (3) noted that the Asia-Pacific region faces a serious epidemic of MetS. In most countries, nearly one-fifth or more of adults are affected by MetS with a long-term increase in prevalence (3). MetS is more widespread in the senior population (4–6). Over 30% of middle-aged and older persons globally had MetS (7), while a research conducted in China in 2020 (8) indicated that 33.38% of the Chinese population had MetS.

In addition, obesity and abdominal fat deposition lead to several metabolic abnormalities that result in increased hepatic glucose output and decreased insulin sensitivity in skeletal muscle, liver, and adipose tissue, processes that are closely linked to the pathogenesis of type 2 diabetes (9). In recent years, the clinical identification of MetS is becoming increasingly important because the MetS has been shown to increase the risk of type 2 diabetes by 5-fold and the risk of cardiovascular disease by 2-fold over 5 to 10 years (10). It is found that the mortality rate of patients with MetS is much higher than that of patients without MetS (11). Furthermore, patients with MetS have a 2- to 4-fold increased risk of stroke, a 3- to 4-fold increased risk of myocardial infarction, and a 2-fold increased risk of dying from such events compared to those without MetS, regardless of a previous history of cardiovascular events (12, 13). Therefore, it is significant to find the best predictor of MetS.

The World Health Organization (WHO) (14), the International Diabetes Federation (IDF) (15), and the NCEP ATP III (2005) (1, 2) have developed some of the more widely used diagnostic criteria for MetS at the moment. However, it can be difficult to use these criteria for diagnosis during extensive screening in primary healthcare facilities. In order to forecast the risk of MetS, there is a clinical need for more simple and user-friendly screening indicators.

Direct assessment of obesity and fat distribution by computed tomography (CT) or magnetic resonance imaging (MRI) is the gold standard (16), but this method is expensive and complex for the general public. The other method is an indirect response to obesity through anthropometric measurements. In recent years, there has been growing epidemiological evidence that simple and easily available anthropometric measures can be used to predict the likelihood of MetS (17, 18). Body mass index (BMI) has been utilized as the most common measure of obesity and overweight in earlier research (19). In a study by Navarra (20), it was proposed that the triglyceride-glucose index(Tyg index) is the best predictor of metabolic disorders. In addition, waist-height ratio (WHtR) (21) was the best indicator of dyslipidemia and hyperglycemia, while waist circumference (WC) was also a better predictor of blood pressure abnormalities. In Iran, the results of a study similarly showed (22) that WHtR has a better diagnostic value for MetS in the elderly, followed by BMI, visceral adiposity index (VAI), waist-to-hip ratio, and neck circumference, with a body shape index (ABSI) having a weaker predictive value (23). Body roundness index (BRI) has been proposed as a potentially useful clinical predictor of MetS in Peruvian adults (24).

The best way to use anthropometric indicators to predict metabolic disease is still up for debate (25), despite the fact that all of these indicators can be used to assess obesity and metabolic disease. This is because anthropometric indicators are influenced by factors like race, age, and gender, and body composition may differ across populations and ethnicities (26–28). The development of programs to alter risk factors and aid in delaying the start and progression of MetS later in life can be aided by the early identification of those who are at risk. Consequently, the goal of this study was to explore the screening and predictive roles of obesity and lipid-related indicators for MetS in middle-aged and elderly Chinese, as well as the optimal predicted cut-off values to offer a basis for MetS prevention and therapy.

## Methods

### Study design and setting

The China Center for Economic Research at Peking University performed the 2011 China Health and Retirement Longitudinal Survey Wave (CHARLS Wave 2011), a nationally representative longitudinal investigation, which provided the data for our study (29). Participants in CHARLS were recruited from May 2011 to March 2012. 9,457 individuals from the CHARLS Wave 2011 study were included in our analysis after missing data subjects were removed. Without any direct interaction with people, all data are provided in the open as microdata at http://charls.pku.edu.cn/index/zh-cn.html. All participants gave their informed consent prior to the collection of data, and the study was approved by the Ethics Committee of the China Center for Economic Research at Peking University.

### Individuals

Study subjects for this investigation were chosen from the China Health and Retirement Longitudinal Study (CHARLS), Wave 1 (2011). The CHARLS Wave 2011 was used to choose participants for this study (29). The average age of the 9457 individuals that participated in CHARLS was 59.28 years (standard deviation SE=9.64, range 45-98 years). Males had a mean age of 60.29 years (SE=9.33; range 45-98 years) while females had a mean age of 58.41 years (SE=9.81; range 45-92 years).

### Baseline characteristics

Baseline characteristics including age, sex, education, marital status, living place, smoking status, drinking status, taking activities, and Having regular exercises, and the counts of Chronic diseases were collected by self-report. Taking activities included communicating with friends or providing help to neighbors, friends, or family, or doing a sport, social, or other kind of club or playing Ma-jong, or playing played cards, chess, or going to community club, or taking part in a community-related organization, or doing voluntary or charity work, or caring for a sick or disabled adult, or attending an educational or training course, or stock investment, or using the Internet were dichotomized as ever (at least once a month) or never (30). Chronic diseases included cancer or malignant tumor, dyslipidemia, hypertension, chronic lung diseases, diabetes or high blood sugar, liver disease, kidney disease, asthma were reported by the respondents, arthritis or rheumatism, stomach or other digestive disease, heart attack, coronary heart disease, angina, congestive heart failure, or other heart problems, emotional, nervous, or psychiatric problems, memory-related disease, stroke (30). Most variables depended on our previous research studies (31–36).

### Definition of MetS

The NCEP ATP III (2005) (1, 2) put forward the definition and diagnostic criteria of MetS. According to the standard Chinese definition (37, 38), components of MetS are divided into five categories (1): the waist circumference of central obesity is defined as ≥ 80 cm for women and ≥ 90 cm for men (2). Elevated TG levels: TG levels ≥ 150 mg/dL (3). Low HDL-C levels: HDL-C levels< 40 mg/dL for men and< 50 mg/dL for women (4). Elevated BP: systolic blood pressure (SBP) ≥130 mmHg and/or diastolic blood pressure (DBP) of ≥ 85 mmHg or using antihypertensive therapy (5). Elevated FPG levels: FPG levels ≥ 100 mg/dL or using antidiabetic medications or self-reported medical history of diabetes. When 3 of 5 of the listed characteristics are present, a diagnosis of metabolic syndrome can be made.

### Glucose, HDL, triglycerides measurement

The Chinese Center for Disease Control and Prevention in Beijing received the venous blood samples within two weeks of them leaving the Centers for Disease Control and Prevention (CDC) station. The samples were immediately stored and frozen at -20°C before being delivered. When the necessary assays were completed in the lab of the Chinese Medical University, they were put in a deep refrigerator and kept at -80°C. At the Capital Medical University Youanmen Clinical Laboratory, triglycerides (TG), fasting plasma glucose (FPG), and high-density lipoprotein cholesterol (HDL-C) were measured using the enzyme colorimetric assay. We divided TG levels into two groups, ≥150 mg/dL and<150 mg/dL, in accordance with a classification that has previously been employed in studies (31). When fasting plasma glucose is ≥126 mg/dL or 7.0 mmol/L and above, it indicates abnormal blood glucose (39). HDL-C values below 40 mg/dL in men and below 50 mg/dL in women were considered abnormal (40).

### Measurements

BMI was calculated by dividing the body weight (kg) by the square of the height (m^2^) (41, 42). During the blood pressure measurement, which is done in a quiet environment with the left arm on a flat surface with the palm facing upward so that the upper arm is at the same height as the heart, the subject should be at ease while sitting. The blood pressure is then measured using a blood pressure monitor. Hypertension was formerly described as having a systolic blood pressure (SBP) greater than 130 mmHg and/or a diastolic blood pressure (DBP) less than 85 mmHg (43, 44). At the conclusion of expiration, the umbilical level was chosen, and the waist size was measured (45); when the waist circumference of women ≥ 80cm, men ≥ 90cm, known as central obesity. WHtR was calculated by the ratio of waist circumference to height (46). VAI was calculated using BMI, WC, TG and HDL-C, with different formulas for men and women (47). It is important to note that VAI, Chinese visceral adiposity index (CVAI), lipid accumulation product index (LAP) and TyG index were required to perform invasive manipulations to obtain TG and HDL-C. Other indicators were calculated using the following equations (48–55).


(1) $$BMI=\frac{Weight}{Height^{2}}$$

(2) $$WHtR=\frac{WC}{Height}$$

(3) $$\begin{array}{l} Males:VAI=\frac{WC}{39.68+(1.88\times BMI)}\times \frac{TG}{1.03}\times \frac{1.31}{HDL}\\ Females:VAI=\frac{WC}{36.58+(1.89\times BMI)}\times \frac{TG}{0.81}\times \frac{1.52}{HDL} \end{array}$$

(4) $$ABSI=\frac{WC}{Height^{\frac{1}{2}}\times BMI^{\frac{2}{3}}}$$

(5) $$BRI=364.2-365.5\sqrt{1-(\frac{WC÷{\left(2\pi \right)}^{2}}{{\left(0.5\times Height\right)}^{2}}}$$

(6) $$\begin{array}{l} Males:LAP=\left[WC\left(cm\right)-65\right]\times TG\left(mmol/l\right)\\ Females:LAP=\left[WC\left(cm\right)-58\right]\times TG\left(mmol/l\right) \end{array}$$

(7) $$CI=\frac{WC(m)}{0.019\sqrt{\frac{weight(kg)}{height(m)}}}$$

(8) $$\begin{array}{l} Males:\\ CVAI=-267.93+0.68\times age+0.03\times BMI\left(kg/m^{2}\right)+4.00\times WC\left(cm\right)+22.00\times Log_{10}TG\left(mmol/l\right)-16.32\times HDL-C\left(mmol/l\right)\\ Females:\\ CVAI=-187.32+1.71\times age+4.32\times BMI\left(kg/m^{2}\right)+1.12\times WC\left(cm\right)+39.76\times Log_{10}TG\left(mmol/l\right)-11.66\times HDL-C\left(mmol/l\right) \end{array}$$

(9) TyGindex=Ln[(TG(mg/dl)×glucose(mg/dl)/2)]

(10) TyG-BMI=TyG×BMI

(11) TyG-WC=TyG×WC

(12) TyG-WHtR=TyG×WHtR

### Statistical analysis

Statistical Product Service Solutions (SPSS) software, version 25.0, was used to conduct the analyses (IBM SPSS, Armonk, NY, USA). By sex, sociodemographic traits were analyzed and percentages were provided. To compare the categorical variable distribution across sex, a chi-square test was utilized. The mean and standard deviation were used to express continuous variables. In order to evaluate the variations in mean distributions by sex, independent samples t-tests were utilized. The unadjusted and adjusted relationships between anthropometric and MetS components were evaluated using binary logistic regression. We calculated odds ratios (ORs) and 95% confidence intervals (95%CI) adjusting for age, sex, educational levels, marital status, live place, current smoking, alcohol drinking, activities, exercises, and chronic diseases. To determine the area under the curve (AUC) and 95% confidence interval as a predictor of MetS, the receiver operating characteristic curve (ROC) was utilized (56). The significance of the AUC is that an area greater than 0.9 indicates high accuracy, 0.7-0.9 indicates moderate accuracy, 0.5-0.7 indicates low accuracy, and 0.5 indicates a chance result (57). The ROC curve can also be used to determine sensitivity, specificity, positive predictive value, negative predictive value, positive likelihood ratio, and negative likelihood ratio. The Youden index, which is derived using the formula: [maximum (sensitivity + specificity-1)] (58), which is the maximum vertical distance between the ROC curve and the diagonal or chance line, determines the cut-off values of the predictor based on the highest value.

## Results

**Table 1** shows the basic characteristics of the participants. A total of 9457 subjects were included in this study, of whom 4340 (45.89%) were male and 5117 (54.11%) were female. Among them, there were significant differences between men and women in age, education, marital status, alcohol consumption, smoking, number of chronic diseases, WC, BMI, WHtR, VAI, ABSI, BRI, LAP, CI, CVAI, TyG index, TyG-BMI, TyG-WC, TyG -WHtR (*P<* 0.05). However, the distribution of residence, social activities, and physical activity habits were not statistically significant between the male and female subgroups (*P* > 0.05). Because of these significant differences between males and females (*P<* 0.05), we performed the main analyses separately by sex.

**Table 1:** Characteristics of participants with full samples (N=9457).

Variables	Male	Female	Total	*P*
	N (%)	N (%)	N (%)	
N	4340 (45.89)	5117 (54.11)	9457 (100)	
Age (years)				
45-54	1272 (29.31)	1919 (37.50)	3191 (33.74)	<0.001
55-64	1719 (39.61)	1924 (37.60)	3643 (38.52)	
65-74	985 (22.70)	898 (17.55)	1883 (19.91)	
≥75	364 (8.39)	376 (7.35)	740 (7.82)	
Education				
Illiterate	593 (13.66)	2176 (42.52)	2769 (29.28)	<0.001
Less than elementary school	3185 (73.39)	2597 (50.75)	5782 (61.14)	
High school	360 (8.29)	253 (4.94)	613 (6.48)	
Above vocational school	202 (4.65)	91 (1.78)	293 (3.10)	
Marital status				
Single	401 (9.24)	769 (15.03)	1170 (12.37)	<0.001
Married	3939 (90.76)	4348 (84.97)	8287 (87.63)	
Current residence				
Rural	3998 (92.12)	4739 (92.61)	8737 (92.39)	0.368
Urban	342 (7.88)	378 (7.39)	720 (7.61)	
Current smoking				
No	1070 (24.65)	4716 (92.16)	5786 (61.18)	<0.001
Former smoke	731 (16.84)	95 (1.86)	826 (8.73)	
Current smoke	2539 (58.50)	306 (5.98)	2845 (30.08)	
Alcohol drinking				
No	1909 (43.99)	4501 (87.96)	6410 (67.78)	<0.001
Less than once a month	470 (10.83)	255 (4.98)	725 (7.67)	
More than once a month	1961 (45.18)	361 (7.05)	2322 (24.55)	
Taking activities				
No	2131 (49.10)	2562 (50.07)	4693 (49.62)	0.349
Yes	2209 (50.90)	2555 (49.93)	4764 (50.38)	
Having regular exercises				
No exercise	2700 (62.21)	3136 (61.29)	5836 (61.71)	0.567
Less than exercises	810 (18.66)	996 (19.46)	1806 (19.10)	
Regular exercises	830 (19.12)	985 (19.25)	1815 (19.19)	
Chronic diseases (counts)				
0	1417 (32.65)	1465 (28.63)	2882 (30.47)	<0.001
01-Feb	2156 (49.68)	2616 (51.12)	4772 (50.46)	
Mar-14	767 (17.67)	1036 (20.25)	1803 (19.07)	
WC	84.97 ± 9.82	85.65 ± 10.16	85.34 ± 10.01	<0.001
BMI	22.96 ± 3.65	23.99 ± 4.05	23.52 ± 3.90	<0.001
WHtR	0.52 ± 0.06	0.56 ± 0.07	0.54 ± 0.07	<0.001
VAI	3.96 ± 4.41	3.96 ± 4.41	3.96 ± 4.41	<0.001
ABSI	8.25 ± 0.53	8.37 ± 0.64	8.32 ± 0.59	<0.001
BRI	3.78 ± 1.14	4.67 ± 1.46	4.26 ± 1.39	<0.001
LAP	30.89 ± 33.35	43.8 ± 35.27	37.87 ± 34.99	<0.001
CI	1.27 ± 0.08	1.27 ± 0.08	1.27 ± 0.08	<0.001
CVAI	95.99 ± 47.56	95.99 ± 47.56	95.99 ± 47.56	<0.001
TyG index	8.62 ± 0.66	8.72 ± 0.63	8.68 ± 0.65	<0.001
TyG-BMI	198.7 ± 39.68	209.85 ± 41.67	204.73 ± 41.14	<0.001
TyG-WC	734.64 ± 118.12	748.87 ± 116.28	742.34 ± 117.34	<0.001
TyG -WHtR	4.48 ± 0.69	4.91 ± 0.76	4.71 ± 0.76	<0.001

**Table 2** shows the baseline characteristics of the study participants with and without MetS by sex. According to the research results, the proportion of women suffering from MetS was much higher (51.83%, compared to 30.67% for men). Men with MetS had significant differences in age, education, current residence, smoking, alcohol consumption, social activity, physical activity, number of chronic diseases, WC, BMI, WHtR, VAI, ABSI, BRI, LAP, CI, CVAI, TyG index, TyG-BMI, TyG-WC and TyG-WHtR (*P<* 0.05); women had significant differences in all aspects (*P<* 0.05), with the exception of education and current residence (*P* > 0.05).

**Table 2:** Baseline characteristics of the study participants with and without MetS by sex.

Variables	Male (N=4340)		*P*	Female (N=5117)		*P*
N (%)	With MetS N (%)	Without MetS N (%)		With MetS N (%)	Without MetS N (%)	
N	1331 (30.67)	3009 (69.33)		2629 (51.38)	2488 (48.62)	
Age (years)						
45-54	412 (30.95)	860 (28.58)	0.017	840 (31.95)	1079 (43.37)	<0.001
55-64	546 (41.02)	1173 (38.98)		1039 (39.52)	885 (35.57)	
65-74	282 (21.19)	703 (23.36)		537 (20.43)	361 (14.51)	
≥75	91 (6.84)	273 (9.07)		213 (8.10)	163 (6.55)	
Education						
Illiterate	141 (10.59)	452 (15.02)	<0.001	1107 (42.11)	1069 (42.97)	0.639
Less than elementary school	976 (73.33)	2209 (73.41)		1343 (51.08)	1254 (50.40)	
High school	126 (9.47)	234 (7.78)		127 (4.83)	126 (5.06)	
Above vocational school	88 (6.61)	114 (3.79)		52 (1.98)	39 (1.57)	
Marital status						
Single	117 (8.79)	284 (9.44)	0.497	412 (15.67)	357 (14.35)	0.186
Married	1214 (91.21)	2725 (90.56)		2217 (84.33)	2131 (85.65)	
Current residence						
Rural	1169 (87.83)	2829 (94.02)	<0.001	2417 (91.94)	2322 (93.33)	0.057
Urban	162 (12.17)	180 (5.98)		212 (8.06)	166 (6.67)	
Current smoking						
No	381 (28.63)	689 (22.90)	<0.001	2409 (91.63)	2307 (92.73)	0.007
Former smoke	284 (21.34)	447 (14.86)		64 (2.43)	31 (1.25)	
Current smoke	666 (50.04)	1873 (62.25)		156 (5.93)	150 (6.03)	
Alcohol drinking						
No	625 (46.96)	1284 (42.67)	0.014	2343 (89.12)	2158 (86.74)	0.031
Less than once a month	148 (11.12)	322 (10.70)		120 (4.56)	135 (5.43)	
More than once a month	558 (41.92)	1403 (46.63)		166 (6.31)	195 (7.84)	
Taking activities						
No	600 (45.08)	1531 (50.88)	<0.001	1249 (47.51)	1313 (52.77)	<0.001
Yes	731 (54.92)	1478 (49.12)		1380 (52.49)	1175 (47.23)	
Having regular exercises						
No exercise	830 (62.36)	1870 (62.15)	0.042	1606 (61.09)	1530 (61.50)	0.006
Less than exercises	224 (16.83)	586 (19.47)		479 (18.22)	517 (20.78)	
Regular exercises	277 (20.81)	553 (18.38)		544 (20.69)	441 (17.73)	
Chronic diseases (counts)						
0	341 (25.62)	1076 (35.76)	<0.001	600 (22.82)	865 (34.77)	<0.001
1-2	660 (49.59)	1496 (49.72)		1350 (51.35)	1266 (50.88)	
3-14	330 (24.79)	437 (14.52)		679 (25.83)	357 (14.35)	
WC	93.29 ± 8.80	81.28 ± 7.79	<0.001	90.31 ± 8.84	80.73 ± 9.09	<0.001
BMI	25.62 ± 3.57	21.79 ± 3.00	<0.001	25.46 ± 3.94	22.44 ± 3.55	<0.001
WHtR	0.56 ± 0.05	0.5 ± 0.05	<0.001	0.59 ± 0.06	0.53 ± 0.06	<0.001
VAI	7.5 ± 6.32	2.4 ± 1.56	<0.001	8.73 ± 6.81	3.28 ± 1.77	<0.001
ABSI	8.38 ± 0.48	8.19 ± 0.54	<0.001	8.48 ± 0.64	8.26 ± 0.62	<0.001
BRI	4.71 ± 1.10	3.37 ± 0.89	<0.001	5.29 ± 1.38	4 ± 1.23	<0.001
LAP	61.21 ± 44.08	17.47 ± 12.57	<0.001	62.44 ± 39.15	24.09 ± 13.54	<0.001
CI	1.32 ± 0.07	1.25 ± 0.08	<0.001	1.33 ± 0.09	1.27 ± 0.09	<0.001
CVAI	143.53 ± 37.06	74.97 ± 34.83	<0.001	133.45 ± 35.65	79.37 ± 32.21	<0.001
TyG index	9.2 ± 0.66	8.37 ± 0.47	<0.001	9.07 ± 0.59	8.36 ± 0.43	<0.001
TyG-BMI	235.7 ± 37.81	182.34 ± 27.66	<0.001	230.95 ± 39.12	187.55 ± 31.41	<0.001
TyG-WC	857.6 ± 99.13	680.25 ± 78.31	<0.001	818.99 ± 97.78	674.78 ± 83.81	<0.001
TyG -WHtR	5.19 ± 0.59	4.17 ± 0.47	<0.001	5.36 ± 0.65	4.43 ± 0.55	<0.001

**Figure 1:**
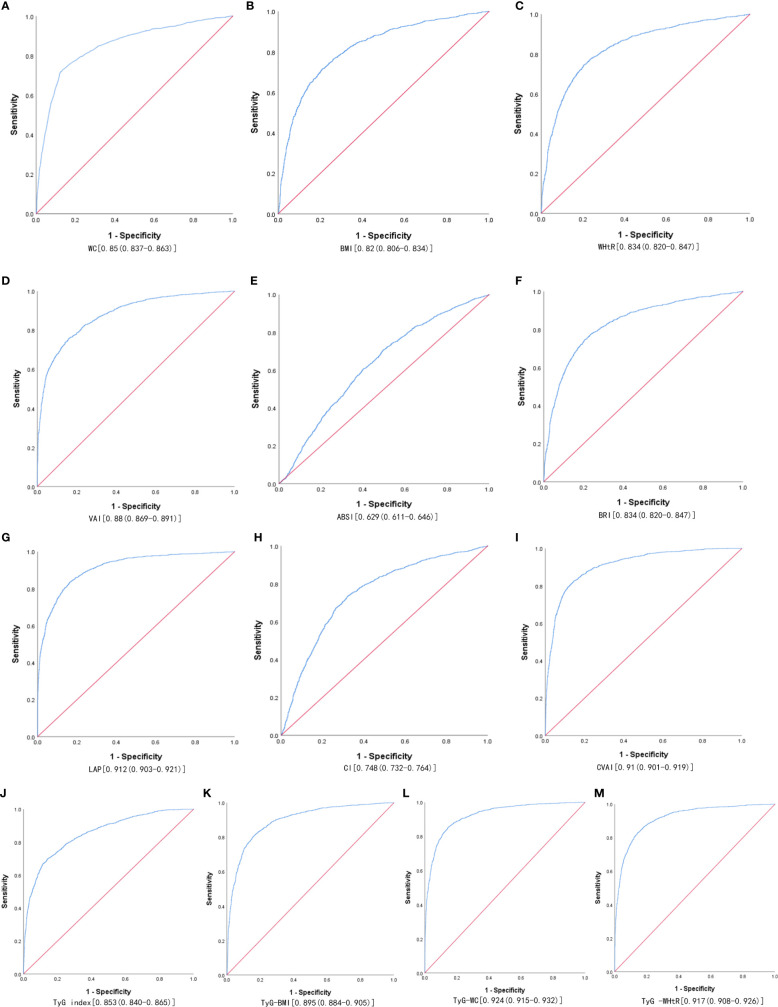
The ROC curves of each indicator in the prediction of MetS risk in males. **(A)** WC, **(B)** BMI, **(C)** WHtR, **(D)** VAI, **(E)** ABSI, **(F)** BRI, **(G)** LAP, **(H)** CI, **(I) **CVAI, **(J)** TyG-index, **(K)** TyG-BMI, **(L)** TyG-WC, **(M)** TyG-WHtR.

**Figure 2:**
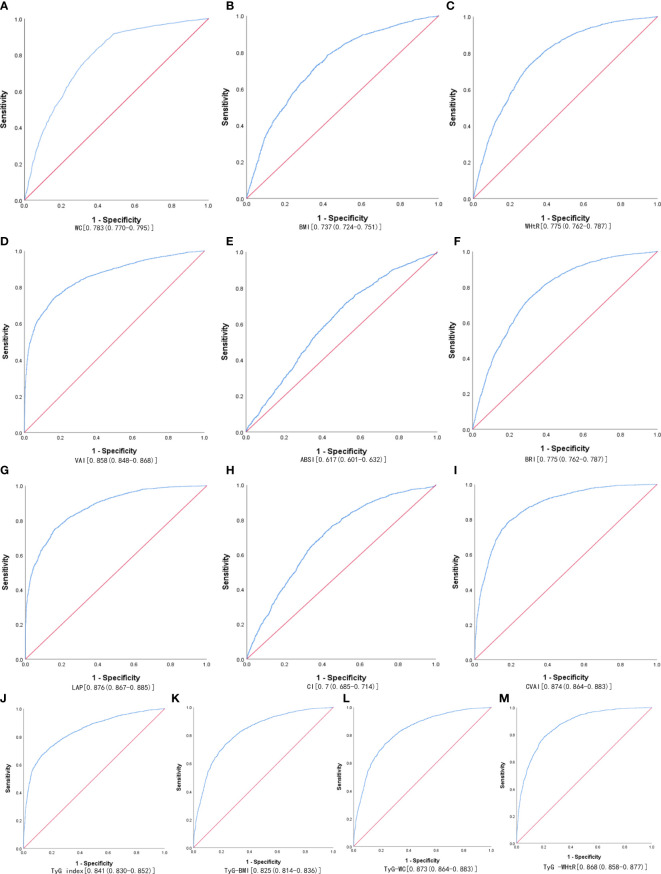
The ROC curves of each indicator in the prediction of MetS risk in females. **(A)** WC, **(B)** BMI, **(C)** WHtR, **(D)** VAI, **(E)** ABSI, **(F)** BRI, **(G)** LAP, **(H)** CI, **(I) **CVAI, **(J)** TyG-index, **(K)** TyG-BMI, **(L)** TyG-WC, **(M)** TyG-WHtR.

**Table 3** shows the cut-off values between the AUC, sensitivity, and specificity for obesity and lipid-related indices to detect MetS by sex. We observed the predictive value of obesity and lipid-related indicators for MetS by sex using the ROC. The ROC curves of each indicator in the prediction of MetS risk in men and women are shown in **Figures 1**, **2** respectively. As shown in the table and figures, among men, the Tyg-WC index was the best predictor of MetS in the middle-aged and elderly male population (AUC=0.924, SE=0.004, 95% CI [0.915,0.932], and optimal cutoffs=763.595). Meanwhile, Tyg-WHtR (AUC=0.917, SE=0.005, 95%CI [0.908,0.926], and optimal cutoffs=4.687) and LAP (AUC=0.912, SE=0.005, 95% CI [0.903,0.921], and optimal cutoffs=27.895) had similar predictive values. Meanwhile, among women, LAP was the most accurate predictor of MetS in middle-aged and elderly women (AUC=0.876, SE=0.005, 95%CI [0.867,0.885], and optimal cutoffs=35.867), CVAI (AUC=0.874, SE=0.005, 95%CI [0.864,0.883], and optimal cutoffs=105.340) and Tyg-WC (AUC=0.873, SE=0.005,95%CI [0.864,0.883], and optimal cutoffs =731.378) had similar predictive values. All of the above indicators were statistically different (*P*<0.05). In addition, the predictive value of ABSI as a predictor was small in both sexes (*P*<0.05).

**Table 3:** Cut-off between AUC, sensitivity and specificity for obesity- and lipid-related indices to detect metabolic syndrome by sex.

N=9457	WC	BMI	WHtR	VAI	ABSI	BRI	LAP	CI	CVAI	TyG index	TyG-BMI	TyG-WC	TyG -WHtR
Male													
AUC	0.850	0.820	0.834	0.880	0.629	0.834	0.912	0.748	0.910	0.853	0.895	0.924	0.917
Std. Error	0.007	0.007	0.007	0.006	0.009	0.007	0.005	0.008	0.005	0.006	0.005	0.004	0.005
95%CI	0.837,0.863	0.806,0.834	0.820,0.847	0.869,0.891	0.611,0.646	0.820,0.847	0.903,0.921	0.732,0.764	0.901,0.919	0.840,0.865	0.884,0.905	0.915,0.932	0.908,0.926
* P*-value	<0.001	<0.001	<0.001	<0.001	<0.001	<0.001	<0.001	<0.001	<0.001	<0.001	<0.001	<0.001	<0.001
Optimal cutoffs	89.850	23.780	0.534	3.643	8.168	4.011	27.895	1.282	111.505	8.840	206.662	763.595	4.687
J-Youden	0.594	0.506	0.542	0.598	0.211	0.542	0.671	0.412	0.681	0.557	0.647	0.709	0.687
Sensitivity (%)	71.60%	71.70%	74.70%	75.50%	70.50%	74.70%	83.50%	73.70%	82.60%	69.80%	81.10%	85.30%	82.90%
Specificity (%)	87.80%	78.90%	79.50%	84.30%	50.60%	79.50%	83.60%	67.50%	85.50%	85.90%	83.60%	85.60%	85.80%
(+) Likelihood ratio	5.869	3.398	3.644	4.814	1.427	3.644	5.091	2.268	5.697	4.950	4.945	5.924	5.853
(-) Likelihood ratio	0.323	0.359	0.318	0.290	0.583	0.318	0.197	0.390	0.204	0.352	0.226	0.172	0.200
Female													
AUC	0.783	0.737	0.775	0.858	0.617	0.775	0.876	0.700	0.874	0.841	0.825	0.873	0.868
Std. Error	0.006	0.007	0.007	0.005	0.008	0.007	0.005	0.007	0.005	0.005	0.006	0.005	0.005
95%CI	0.770,0.795	0.724,0.751	0.762,0.787	0.848,0.868	0.601,0.632	0.762,0.787	0.867,0.885	0.685,0.714	0.864,0.883	0.830,0.852	0.814,0.836	0.864,0.883	0.858,0.877
* P*-value	<0.001	<0.001	<0.001	<0.001	<0.001	<0.001	<0.001	<0.001	<0.001	<0.001	<0.001	<0.001	<0.001
Optimal cutoffs	82.150	22.706	0.555	4.563	8.172	4.445	35.867	1.275	105.340	8.811	206.959	731.378	4.834
J-Youden	0.439	0.364	0.430	0.575	0.194	0.430	0.588	0.313	0.602	0.539	0.509	0.585	0.580
Sensitivity (%)	83.00%	78.50%	73.20%	75.00%	71.60%	73.20%	75.40%	76.20%	78.60%	66.80%	73.50%	81.40%	78.70%
Specificity (%)	60.90%	57.90%	69.80%	82.50%	47.80%	69.80%	83.40%	55.10%	81.60%	87.10%	77.40%	77.10%	79.30%
(+) Likelihood ratio	2.123	1.865	2.424	4.286	1.372	2.424	4.542	1.697	4.272	5.178	3.252	3.555	3.802
(-) Likelihood ratio	0.279	0.371	0.384	0.303	0.594	0.384	0.295	0.432	0.262	0.381	0.342	0.241	0.269

WC, waist circumference; BMI, body mass index; WHtR, waist to height ratio; VAI, visceral adiposity index; ABSI, A body shape index; BRI, body roundness index; LAP, lipid accumulation product; CVAI, Chinese visceral adiposity index; CI, conicity index; TyG, triglyceride and glucose index; TyG-BMI, TyG related to BMI; TyG-WC, TyG related to WC; TyG-WHtR, TyG related to WHtR.

**Figure 3:**
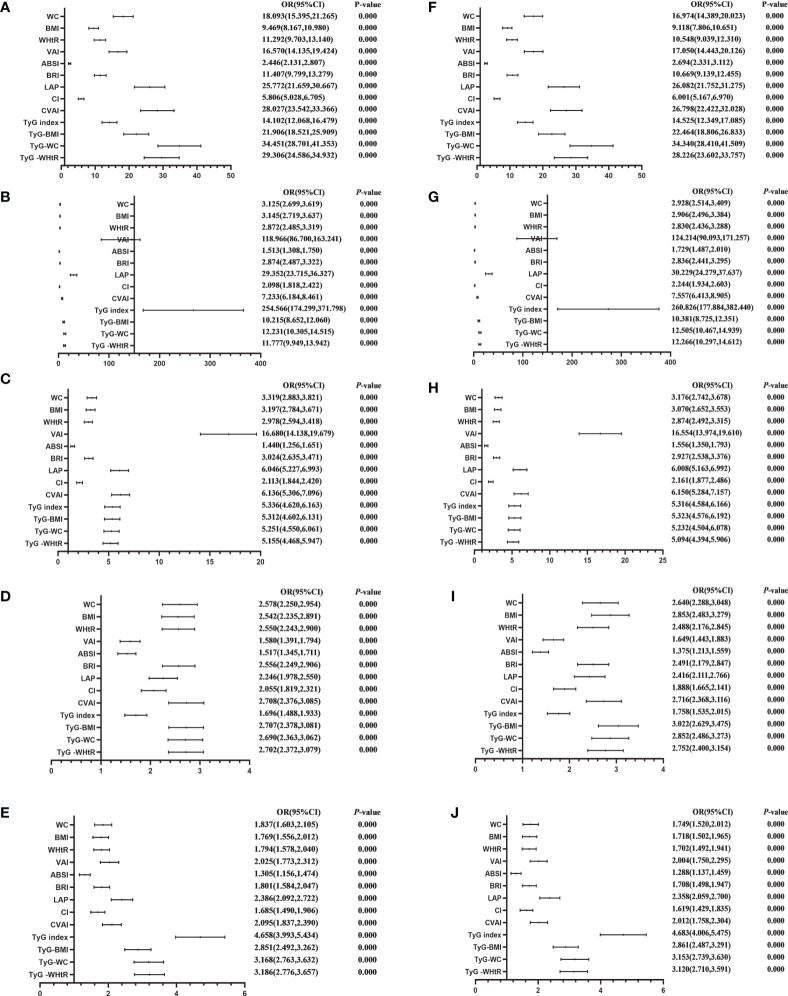
Forest diagram of OR before and after adjustment of confounding factors for male. **(A)** MetS unadjusted, **(B)** Elevated triglycerides unadjusted, **(C)** Reduced HDL-C unadjusted, **(D)** Elevated blood pressure unadjusted, **(E)** Elevated fasting glucose unadjusted, **(F)** MetS adjusted, **(G)** Elevated triglycerides adjusted, **(H)** Reduced HDL-C adjusted, **(I)** Elevated blood pressure adjusted, **(J)** Elevated fasting glucose adjusted. Adjusted OR: Adjusted for age, educational levels, marital status, live place, current smoking, alcohol drinking, activities, exercises, chronic diseases.

**Figure 4:**
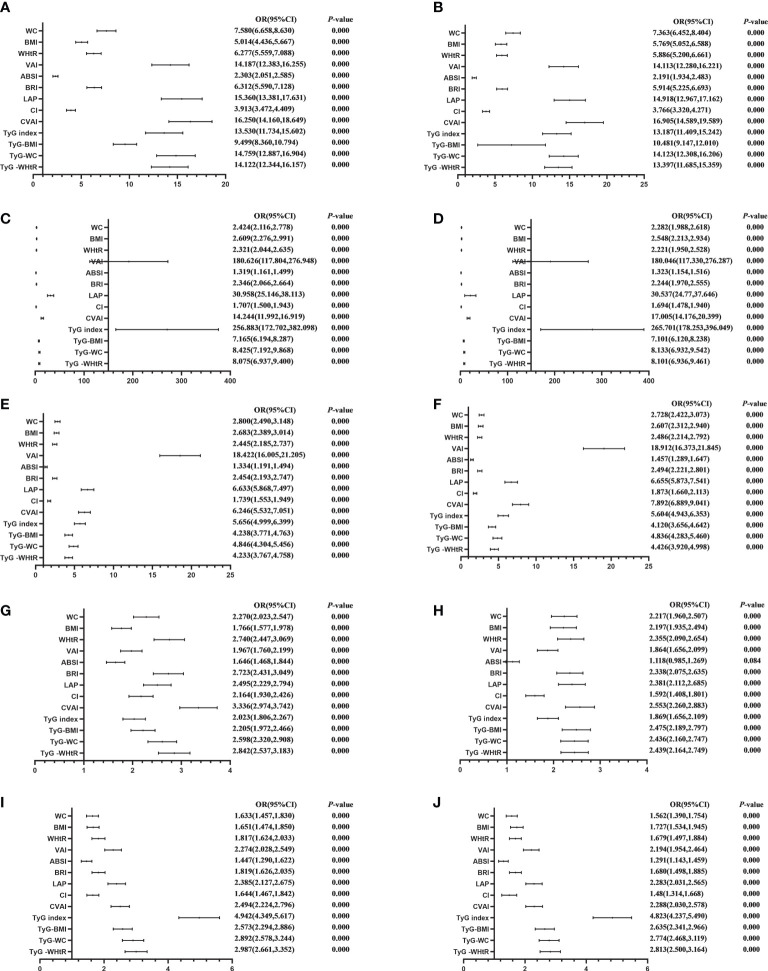
Forest diagram of OR before and after adjustment of confounding factors for female. **(A)** MetS unadjusted, **(B)** Elevated triglycerides unadjusted, **(C)** Reduced HDL-C unadjusted, **(D)** Elevated blood pressure unadjusted, **(E)** Elevated fasting glucose unadjusted, **(F)** MetS adjusted, **(G)** Elevated triglycerides adjusted, **(H)** Reduced HDL-C adjusted, **(I)** Elevated blood pressure adjusted, **(J)** Elevated fasting glucose adjusted. Adjusted OR: Adjusted for age, educational levels, marital status, live place, current smoking, alcohol drinking, activities, exercises, chronic diseases.

**Table 4** shows the associations of obesity- and lipid-related indices with MetS and its components. According to the values in **Table 3**, 13 obesity- and lipid-related indices were transformed into two-category variables in this investigation. **Table 4** is based on the transformed variables. A larger OR, in general, suggests a higher risk factor. Both before and after adjusting for age, education, marital status, current residence, current smoking, alcohol drinking, social activity, exercise, and chronic disease counts, the odds of MetS increased progressively with increasing obesity and units of lipid measurement for both men and women. Each unit rise in the Tyg index, for example, was related to a 14.525-fold increase in the likelihood of developing MetS in males(95% CI: 12.349,17.085). Each unit increase in BMI was linked to a 5.769-fold increase in the likelihood of developing MetS in women(95% CI: 5.052,6.588). In addition, 13 indicators had statistical significance after adjustment of confounding factors (*P* < 0.05). **Figures 3**, **4** show the forest diagram of or value before and after adjustment of confounding factors for males and females, respectively.

**Table 4:** Associations of obesity- and lipid-related indices with MetS and its components.

MetS components	WC	BMI	WHtR	VAI	ABSI	BRI	LAP	CI	CVAI	TyG index	TyG-BMI	TyG-WC	TyG -WHtR
Male													
MetS													
Unadjusted OR (95% CI)	18.093 (15.395,21.265)	9.469 (8.167,10.98)	11.292 (9.703,13.140)	16.570 (14.135,19.424)	2.446 (2.131,2.807)	11.407 (9.799,13.279)	25.772 (21.659,30.667)	5.806 (5.028,6.705)	28.027 (23.542,33.366)	14.102 (12.068,16.479)	21.906 (18.521,25.909)	34.451 (28.701,41.353)	29.306 (24.586,34.932)
*P* value	0.000	0.000	0.000	0.000	0.000	0.000	0.000	0.000	0.000	0.000	0.000	0.000	0.000
Adjusted OR (95% CI)	16.974 (14.389,20.023)	9.118 (7.806,10.651)	10.548 (9.039,12.31)	17.050 (14.443,20.126)	2.694 (2.331,3.112)	10.669 (9.139,12.455)	26.082 (21.752,31.275)	6.001 (5.167,6.97)	26.798 (22.422,32.028)	14.525 (12.349,17.085)	22.464 (18.806,26.833)	34.340 (28.41,41.509)	28.226 (23.602,33.757)
*P* value	0.000	0.000	0.000	0.000	0.000	0.000	0.000	0.000	0.000	0.000	0.000	0.000	0.000
Elevated triglycerides													
Unadjusted OR (95% CI)	3.125 (2.699,3.619)	3.145 (2.719,3.637)	2.872 (2.485,3.319)	118.966 (86.7,163.241)	1.513 (1.308,1.750)	2.874 (2.487,3.322)	29.352 (23.715,36.327)	2.098 (1.818,2.422)	7.233 (6.184,8.461)	254.566 (174.299,371.798)	10.215 (8.652,12.060)	12.231 (10.305,14.515)	11.777 (9.949,13.942)
*P* value	0.000	0.000	0.000	0.000	0.000	0.000	0.000	0.000	0.000	0.000	0.000	0.000	0.000
Adjusted OR (95% CI)	2.928 (2.514,3.409)	2.906 (2.496,3.384)	2.83 (2.436,3.288)	124.214 (90.093,171.257)	1.729 (1.487,2.010)	2.836 (2.441,3.295)	30.229 (24.279,37.637)	2.244 (1.934,2.603)	7.557 (6.413,8.905)	260.826 (177.884,382.440)	10.381 (8.725,12.351)	12.505 (10.467,14.939)	12.266 (10.297,14.612)
*P* value	0.000	0.000	0.000	0.000	0.000	0.000	0.000	0.000	0.000	0.000	0.000	0.000	0.000
Reduced HDL-C													
Unadjusted OR (95% CI)	3.319 (2.883,3.821)	3.197 (2.784,3.671)	2.978 (2.594,3.418)	16.680 (14.138,19.679)	1.440 (1.256,1.651)	3.024 (2.635,3.471)	6.046 (5.227,6.993)	2.113 (1.844,2.420)	6.136 (5.306,7.096)	5.336 (4.620,6.163)	5.312 (4.602,6.131)	5.251 (4.550,6.061)	5.155 (4.468,5.947)
*P* value	0.000	0.000	0.000	0.000	0.000	0.000	0.000	0.000	0.000	0.000	0.000	0.000	0.000
Adjusted OR (95% CI)	3.176 (2.742,3.678)	3.070 (2.652,3.553)	2.874 (2.492,3.315)	16.554 (13.974,19.610)	1.556 (1.350,1.793)	2.927 (2.538,3.376)	6.008 (5.163,6.992)	2.161 (1.877,2.486)	6.150 (5.284,7.157)	5.316 (4.584,6.166)	5.323 (4.576,6.192)	5.232 (4.504,6.078)	5.094 (4.394,5.906)
*P* value	0.000	0.000	0.000	0.000	0.000	0.000	0.000	0.000	0.000	0.000	0.000	0.000	0.000
Elevated blood pressure													
Unadjusted OR (95% CI)	2.578 (2.250,2.954)	2.542 (2.235,2.891)	2.550 (2.243,2.900)	1.580 (1.391,1.794)	1.517 (1.345,1.711)	2.556 (2.249,2.906)	2.246 (1.978,2.550)	2.055 (1.819,2.321)	2.708 (2.376,3.085)	1.696 (1.488,1.933)	2.707 (2.378,3.081)	2.690 (2.363,3.062)	2.702 (2.372,3.079)
*P* value	0.000	0.000	0.000	0.000	0.000	0.000	0.000	0.000	0.000	0.000	0.000	0.000	0.000
Adjusted OR (95% CI)	2.640 (2.288,3.048)	2.853 (2.483,3.279)	2.488 (2.176,2.845)	1.649 (1.443,1.883)	1.375 (1.213,1.559)	2.491 (2.179,2.847)	2.416 (2.111,2.766)	1.888 (1.665,2.141)	2.716 (2.368,3.116)	1.758 (1.535,2.015)	3.022 (2.629,3.475)	2.852 (2.486,3.273)	2.752 (2.400,3.154)
*P* value	0.000	0.000	0.000	0.000	0.000	0.000	0.000	0.000	0.000	0.000	0.000	0.000	0.000
Elevated fasting glucose													
Unadjusted OR (95% CI)	1.837 (1.603,2.105)	1.769 (1.556,2.012)	1.794 (1.578,2.040)	2.025 (1.773,2.312)	1.305 (1.156,1.474)	1.801 (1.584,2.047)	2.386 (2.092,2.722)	1.685 (1.490,1.906)	2.095 (1.837,2.390)	4.658 (3.993,5.434)	2.851 (2.492,3.262)	3.168 (2.763,3.632)	3.186 (2.776,3.657)
*P* value	0.000	0.000	0.000	0.000	0.000	0.000	0.000	0.000	0.000	0.000	0.000	0.000	0.000
Adjusted OR (95% CI)	1.749 (1.520,2.012)	1.718 (1.502,1.965)	1.702 (1.492,1.941)	2.004 (1.750,2.295)	1.288 (1.137,1.459)	1.708 (1.498,1.947)	2.358 (2.059,2.700)	1.619 (1.429,1.835)	2.012 (1.758,2.304)	4.683 (4.006,5.475)	2.861 (2.487,3.291)	3.153 (2.739,3.630)	3.120 (2.710,3.591)
*P* value	0.000	0.000	0.000	0.000	0.000	0.000	0.000	0.000	0.000	0.000	0.000	0.000	0.000
Female													
MetS													
Unadjusted OR (95% CI)	7.580 (6.658,8.630)	5.014 (4.436,5.667)	6.277 (5.559,7.088)	14.187 (12.383,16.255)	2.303 (2.051,2.585)	6.312 (5.590,7.128)	15.360 (13.381,17.631)	3.913 (3.472,4.409)	16.250 (14.160,18.649)	13.530 (11.734,15.602)	9.499 (8.360,10.794)	14.759 (12.887,16.904)	14.122 (12.344,16.157)
*P* value	0.000	0.000	0.000	0.000	0.000	0.000	0.000	0.000	0.000	0.000	0.000	0.000	0.000
Adjusted OR (95% CI)	7.363 (6.452,8.404)	5.769 (5.052,6.588)	5.886 (5.200,6.661)	14.113 (12.280,16.221)	2.191 (1.934,2.483)	5.914 (5.225,6.693)	14.918 (12.967,17.162)	3.766 (3.320,4.271)	16.905 (14.589,19.589)	13.187 (11.409,15.242)	10.481 (9.147,12.010)	14.123 (12.308,16.206)	13.397 (11.685,15.359)
*P* value	0.000	0.000	0.000	0.000	0.000	0.000	0.000	0.000	0.000	0.000	0.000	0.000	0.000
Elevated triglycerides													
Unadjusted OR (95% CI)	2.424 (2.116,2.778)	2.609 (2.276,2.991)	2.321 (2.044,2.635)	180.626 (117.804,276.948)	1.319 (1.161,1.499)	2.346 (2.066,2.664)	30.958 (25.146,38.113)	1.707 (1.500,1.943)	14.244 (11.992,16.919)	256.883 (172.702,382.098)	7.165 (6.194,8.287)	8.425 (7.192,9.868)	8.075 (6.937,9.400)
*P* value	0.000	0.000	0.000	0.000	0.000	0.000	0.000	0.000	0.000	0.000	0.000	0.000	0.000
Adjusted OR (95% CI)	2.282 (1.988,2.618)	2.548 (2.213,2.934)	2.221 (1.950,2.528)	180.046 (117.330,276.287)	1.323 (1.154,1.516)	2.244 (1.970,2.555)	30.537 (24.77,37.646)	1.694 (1.478,1.940)	17.005 (14.176,20.399)	265.701 (178.253,396.049)	7.101 (6.120,8.238)	8.133 (6.932,9.542)	8.101 (6.936,9.461)
*P* value	0.000	0.000	0.000	0.000	0.000	0.000	0.000	0.000	0.000	0.000	0.000	0.000	0.000
Reduced HDL-C													
Unadjusted OR (95% CI)	2.800 (2.490,3.148)	2.683 (2.389,3.014)	2.445 (2.185,2.737)	18.422 (16.005,21.205)	1.334 (1.191,1.494)	2.454 (2.193,2.747)	6.633 (5.868,7.497)	1.739 (1.553,1.949)	6.246 (5.532,7.051)	5.656 (4.999,6.399)	4.238 (3.771,4.763)	4.846 (4.304,5.456)	4.233 (3.767,4.758)
*P* value	0.000	0.000	0.000	0.000	0.000	0.000	0.000	0.000	0.000	0.000	0.000	0.000	0.000
Adjusted OR (95% CI)	2.728 (2.422,3.073)	2.607 (2.312,2.940)	2.486 (2.214,2.792)	18.912 (16.373,21.845)	1.457 (1.289,1.647)	2.494 (2.221,2.801)	6.655 (5.873,7.541)	1.873 (1.660,2.113)	7.892 (6.889,9.041)	5.604 (4.943,6.353)	4.120 (3.656,4.642)	4.836 (4.283,5.460)	4.426 (3.920,4.998)
*P* value	0.000	0.000	0.000	0.000	0.000	0.000	0.000	0.000	0.000	0.000	0.000	0.000	0.000
Elevated blood pressure													
Unadjusted OR (95% CI)	2.270 (2.023,2.547)	1.766 (1.577,1.978)	2.740 (2.447,3.069)	1.967 (1.760,2.199)	1.646 (1.468,1.844)	2.723 (2.431,3.049)	2.495 (2.229,2.794)	2.164 (1.930,2.426)	3.336 (2.974,3.742)	2.023 (1.806,2.267)	2.205 (1.972,2.466)	2.598 (2.320,2.908)	2.842 (2.537,3.183)
*P* value	0.000	0.000	0.000	0.000	0.000	0.000	0.000	0.000	0.000	0.000	0.000	0.000	0.000
Adjusted OR (95% CI)	2.217 (1.960,2.507)	2.197 (1.935,2.494)	2.355 (2.090,2.654)	1.864 (1.656,2.099)	1.118 (0.985,1.269)	2.338 (2.075,2.635)	2.381 (2.112,2.685)	1.592 (1.408,1.801)	2.553 (2.260,2.883)	1.869 (1.656,2.109)	2.475 (2.189,2.797)	2.436 (2.160,2.747)	2.439 (2.164,2.749)
*P* value	0.000	0.000	0.000	0.000	0.084	0.000	0.000	0.000	0.000	0.000	0.000	0.000	
Elevated fasting glucose													
Unadjusted OR (95% CI)	1.633 (1.457,1.830)	1.651 (1.474,1.850)	1.817 (1.624,2.033)	2.274 (2.028,2.549)	1.447 (1.290,1.622)	1.819 (1.626,2.035)	2.385 (2.127,2.675)	1.644 (1.467,1.842)	2.494 (2.224,2.796)	4.942 (4.349,5.617)	2.573 (2.294,2.886)	2.892 (2.578,3.244)	2.987 (2.661,3.352)
*P* value	0.000	0.000	0.000	0.000	0.000	0.000	0.000	0.000	0.000	0.000	0.000	0.000	0.000
Adjusted OR (95% CI)	1.562 (1.390,1.754)	1.727 (1.534,1.945)	1.679 (1.497,1.884)	2.194 (1.954,2.464)	1.291 (1.143,1.459)	1.680 (1.498,1.885)	2.283 (2.031,2.565)	1.48 (1.314,1.668)	2.288 (2.030,2.578)	4.823 (4.237,5.490)	2.635 (2.341,2.966)	2.774 (2.468,3.119)	2.813 (2.500,3.164)
*P* value	0.000	0.000	0.000	0.000	0.000	0.000	0.000	0.000	0.000	0.000	0.000	0.000	0.000

WC, waist circumference; BMI, body mass index; WHtR, waist to height ratio; VAI, visceral adiposity index; ABSI, A body shape index; BRI, body roundness index; LAP, lipid accumulation product; CVAI, Chinese visceral adiposity index; CI, conicity index; TyG, triglyceride and glucose index; TyG-BMI, TyG related to BMI; TyG-WC, TyG related to WC; TyG-WHtR, TyG related to WHtR Odds ratios were adjusted for age, sex, educational levels, marital status, live place, current smoking, alcohol drinking, activities, exercises, chronic diseases.

## Discussion

A total of 9457 middle-aged and elderly Chinese were included in this study, and the overall prevalence of the study population was 41.87% according to the diagnostic criteria of NCEP ATP III. Among them, there were 4340 males and 30.67% were diagnosed with MetS. There were 5117 females, and 51.38% were diagnosed with MetS. It is easy to see that the prevalence of MetS is high in our middle-aged and elderly population, both in men and women. Therefore, it is necessary to explore indicators that can predict the prevalence in order to prevent and delay the increase of prevalence.

In this cross-sectional study, 12 indicators were found to have much higher predictive value for MetS than ABSI. In a similar vein, it has been demonstrated (23, 24, 59–61) that although ABSI is linked to insulin resistance in older Chinese adults without diabetes and in general Chinese adults, it does not correlate well with various risks and MetS and is, therefore, a less desirable insulin resistance discriminator. This result is due to the high concentration of ABSI near the average value and relatively small variance, which makes ABSI perform poorly in predicting chronic diseases (62). The predictive power of Tyg-related parameters was more prominent in both sexes. In addition, LAP and CVAI are also good at predicting MetS.

Firstly, the excellent predictive power of Tyg-related parameters has to be mentioned first. In this study, the predictive ability of the TYG-related parameter (Tyg index, Tyg-WC, Tyg-BMI, and Tyg-WhtR) was excellent in both sexes(*P* < 0.05). A notion initially introduced by Ko et al. (63) is known as the Tyg index-related parameters, which integrate the TyG index with WC, BMI, and WHtR. They discovered that measures associated with the TyG index had the highest AUC values for predicting IR, compared to lipid measurements, lipid ratios, visceral obesity indicators, and adipokines. It was demonstrated in a cross-sectional research by Raimi et al. (64) that the TyG index was useful in diagnosing MetS and that the combination of the TyG index and anthropometric characteristics enhanced MetS identification and prediction; similarly, our findings demonstrated that WC, BMI, and WHtR alone were all less predictive of MetS than when combined with Tyg index predictive power of the pertinent parameters (Tyg-WC, Tyg-BMI, and Tyg-WhtR). And the findings of earlier researchers support our findings (63, 65–68), but these studies involved fewer participants.

Secondly, we found that although the prediction value of LAP is good, and the cut-off values obtained in our research were slightly different from that of other scholars. The cut-off values of LAP for predicting MetS in our study were 27.895 cm·mmol/L in men and 35.867 cm·mmol/L in women. But the results in other studies are different. For example, Haijiang Dai et al. (69) found that MetS prevalence increased with increasing LAP levels in both male and female groups. Maximum sensitivity and specificity for the diagnosis of MetS were found when LAP ≥ 30.5 cm·mmol/L in men and LAP ≥ 23.0 cm·mmol/L in women. In a study by Nascimento-Ferreira et al. (70), the greatest sensitivity and specificity were obtained with LAP cut-off values of 34.2 cm·mmol/L for the total sample. Stratification by age yielded LAP cut-off values of 64.1 cm.mmol/L and 38 cm.mmol/L in males and females under 50 years of age, respectively; for subjects over 50 years of age, the LAP cut-off values were 36.4 cm.mmol/L and 34.2 cm·mmol/L in males and females, respectively. The different LAP metrics in males and females’ performance may be due to the physiological differences in height, weight, and body composition between the sexes, with adipose tissue more likely to accumulate around the trunk and abdomen in men and usually around the hips and thighs in women (71). LAP is closely related to the development of MetS, and several studies at home and abroad have affirmed the value of LAP in predicting and diagnosing MetS (72–75), but the LAP had some differences in the cut-off values, and the reasons for this may be related to many factors such as race, differences in the mean age of the study subjects and the different definitions of MetS chosen.

Thirdly, Chinese Visceral Adiposity Index (CVAI) (51) was put forward because there are racial restrictions in the measurement of VAI, and it has been proved that CVAI is more suitable for China people’s physique (76). The ability of CVAI in predicting MetS is obviously superior to traditional indicators (BMI, WC, etc.) (77, 78). In our study, after adjusting for confounding factors such as sex, age and education, the probability of male patients with high CVAI is still high (adjusted OR=26.798). Therefore, CVAI can also be considered as an independent factor of the prevalence of MetS.

Limitations in this study should be aware of. Firstly, because our study was cross-sectional in nature, we were unable to determine a temporal relationship between adiposity measurements and MetS. Future research must evaluate the association between anthropometric measurements and MetS risk throughout time and develop clinical tools, such as cut-off values, to forecast MetS risk. Secondly, even though confounders were taken into account in the multivariate regression model, this link should be investigated prospectively since unmeasured or inaccurately measured covariates may lead to residual confounding. Thirdly, many participants were excluded because of the lack of data, and further research should collect more complete data. The study’s very large sample of 9457 middle-aged and older Chinese is another important strength. The analytical approach that managed the various confounders is another strength.

## Conclusions

Among the middle-aged and elderly population in China, after adjusting for confounding factors, all the indicators except ABSI had good predictive power. The predictive power of Tyg-related parameters was more prominent in both sexes. In addition, LAP and CVAI are also good at predicting MetS.

## Data availability statement

The original contributions presented in the study are included in the article/supplementary materials. Further inquiries can be directed to the corresponding author.

## Ethics statement

Approval for this study was given by the medical ethics committee of Wannan medical college (approval number 2021–3). The patients/participants provided their written informed consent to participate in this study.

## Author contributions

Conceived and designed the research: LZ. Wrote the paper: YQL. Analyzed the data: YQL and LZ. Revised the paper: YQL, JG, LZ, HL, L-LG, JLL, YXL, XL, LS, LY, TY, CW, DZ, HW, JL, ML, and YH. The authors read and approved the final manuscript.

## Glossary

**Table:**

CHARLS	China Health and Retirement Longitudinal Study
WC	waist circumference
BMI	body mass index
WHtR	waist-height ratio
VAI	visceral adiposity index
ABSI	a body shape index
BRI	body roundness index
LAP	lipid accumulation product index
CI	conicity index
CVAI	Chinese visceral adiposity index
TyG-index	triglyceride-glucose index
ROC	receiver operating characteristic curve
AUC	area under curve
MetS	Metabolic syndrome
HDL-C	high-density lipoprotein cholesterol
WHO	The World Health Organization
IDF	the International Diabetes Federation
NCEP ATP III	National Cholesterol Education Program Adult Treatment Panel III
CDS	the Chinese Medical Association Diabetes Society
CT	Computed Tomography
MRI	magnetic resonance imaging
BP	blood pressure
SBP	systolic blood pressure
DBP	diastolic blood pressure
TG	triglycerides
FPG	fasting plasma glucose
CDC	Centers for Disease Control and Prevention
SPSS	Statistical Product Service Solutions
ORs	odds ratios
CI	confidence interval
SE	standard error
